# Mixture models detect large effect QTL better than GBLUP and result in more accurate and persistent predictions

**DOI:** 10.1186/s40104-016-0066-z

**Published:** 2016-02-11

**Authors:** Anna Wolc, Jesus Arango, Petek Settar, Janet E. Fulton, Neil P. O’Sullivan, Jack C. M. Dekkers, Rohan Fernando, Dorian J. Garrick

**Affiliations:** Department of Animal Science, Iowa State University, 225D Kildee Hall, Ames, IA 50011 USA; Hy-Line International, Dallas Center, IA USA

**Keywords:** Bayesian methods, Bias, QTL

## Abstract

**Background:**

Accurate evaluation of SNP effects is important for genome wide association studies and for genomic prediction. The genetic architecture of quantitative traits differs widely, with some traits exhibiting few if any quantitative trait loci (QTL) with large effects, while other traits have one or several easily detectable QTL with large effects.

**Methods:**

Body weight in broilers and egg weight in layers are two examples of traits that have QTL of large effect. A commonly used method for genome wide association studies is to fit a mixture model such as BayesB that assumes some known proportion of SNP effects are zero. In contrast, the most commonly used method for genomic prediction is known as GBLUP, which involves fitting an animal model to phenotypic data with the variance-covariance or genomic relationship matrix among the animals being determined by genome wide SNP genotypes. Genotypes at each SNP are typically weighted equally in determining the genomic relationship matrix for GBLUP. We used the equivalent marker effects model formulation of GBLUP for this study. We compare these two classes of models using egg weight data collected over 8 generations from 2,324 animals genotyped with a 42 K SNP panel.

**Results:**

Using data from the first 7 generations, both BayesB and GBLUP found the largest QTL in a similar well-recognized QTL region, but this QTL was estimated to account for 24 % of genetic variation with BayesB and less than 1 % with GBLUP. When predicting phenotypes in generation 8 BayesB accounted for 36 % of the phenotypic variation and GBLUP for 25 %. When using only data from any one generation, the same QTL was identified with BayesB in all but one generation but never with GBLUP. Predictions of phenotypes in generations 2 to 7 based on only 295 animals from generation 1 accounted for 10 % phenotypic variation with BayesB but only 6 % with GBLUP. Predicting phenotype using only the marker effects in the 1 Mb region that accounted for the largest effect on egg weight from generation 1 data alone accounted for almost 8 % variation using BayesB but had no predictive power with GBLUP.

**Conclusions:**

In conclusion, In the presence of large effect QTL, BayesB did a better job of QTL detection and its genomic predictions were more accurate and persistent than those from GBLUP.

## Background

The choice of methods for accurate evaluation of SNP effects in the presence of large effect QTL can be critical for genome wide association studies (GWAS) and for genomic selection (GS). Mixture models, sometimes known as variable selection models, assume some SNP effects are zero. One such mixture model, method BayesB [1], has been shown to adequately estimate marker effects in simulated data [2, 3] but comparisons based on real data where true effects are unknown are less straightforward. In a recent paper using broiler body weight data, Wang et al. [4] conclude that compared to single step GBLUP procedures “BayesB appears to overly shrink regions to zero, while overestimating the amount of genetic variation attributed to the remaining SNP effects”. Egg weight is one trait in layers for which multiple studies report detecting a QTL with large effect [5–8] at a consistent position on chromosome 4. The objective of this study was to demonstrate that in the presence of such large effect QTL, mixture models like BayesB and BayesC [9, 10] are superior to GBLUP for detecting and quantifying the effects of QTL, and that mixture models provide more accurate genomic estimates of breeding values (GEBV) that are more persistent over generations, using brown egg layer data.

## Methods

Average egg weight data of 3–5 eggs collected at 42–45 wk of age from a brown egg layer line, described in detail by Wolc et al. [8], were used. Only records of animals that were genotyped and had individual performance data were retained, resulting in the following numbers of observations in each of 8 consecutive generations: 295, 323, 294, 360, 290, 252, 300 and 210. All 2,324 animals were genotyped with a custom made 42 K Illumina SNP chip, of which 24,383 segregating SNPs were retained after quality control (removing SNPs with minor allele frequency <0.025, proportion of missing genotypes >0.05, and parent-offspring mismatches >0.05). Build WUGSC 2.1/galGal3 (http://moma.ki.au.dk/genome-mirror/cgi-bin/hgGateway?db=galGal3) was used to identify the genomic locations of markers. Data were analyzed using mixture models that assume π = 99 % of SNPs have no effect, namely BayesB [1], which assumes a different unknown variance for each SNP, BayesC [9, 10], which assumes equal unknown variance for all SNPs. Performance of the mixture models were compared to two ridge regression methods that fit all SNPs in the model (i.e. π = 0), assuming equal variance of SNP effects. The latter methods were two parameterizations of BayesC0, one with the same degrees of freedom (4, 10) for the genetic and residual scale factors as used for BayesB and BayesC, which results in the variance components being estimated jointly from the data and the prior, and one with large values for the degrees of freedom for the genetic and residual scale factor priors (100, 100), which is equivalent to GBLUP [11] in treating the equal variance of SNP effects as known. This model was referred to as GBLUP throughout the paper. The fixed class effect of hatch within generation was fitted in addition to random marker effects. All methods were implemented in the GenSel software [12], which uses Gibbs sampling MCMC algorithms. For inference on parameters we used a chain of 35,000 samples with the first 5,000 discarded as burn in. As a reference for accuracy of prediction, pedigree-based analysis was used with the phenotypes of genotyped individuals and their pedigree which included 26,300 individuals from generations −2 (2 generations prior to the first generation with data) to generation 8.

The marker effects were estimated using whole genome data from all of the first 7 generations, as well as from each generation separately. Accuracy of prediction was assessed as the correlation between GEBV and hatch-corrected phenotype in the validation generation, which was generation 8, or in each successive generation when training only used the 295 individuals from generation 1. Accuracy of prediction was quantified using marker effects from the whole genome, or using only estimates of effects of markers located in the largest QTL region, which was defined as the 1 Mb window that accounted for the largest proportion of variance (the effects of the 1 Mb markers were extracted from solutions of a model fitting all SNPs simultaneously). Persistence of predictions was quantified in terms of the correlations in successive post-training generations.

In order to quantify potential bias in the estimation of the largest effect by BayesB, the SNP that explained the greatest proportion of variance in the 1 Mb window that contributed the largest proportion of variance was fitted as a fixed effect in an animal model using ASReml [13] and the regression coefficients for predicting the 8^th^ generation of hatch-corrected phenotypes were calculated and compared to the corresponding regression from BayesB – a regression coefficient of phenotype on the SNP effect estimate greater than 1 would indicate overshrinkage of the SNP effect; this means that the observed difference in phenotypes is larger than expected based on marker solutions.

## Results and discussion

### Detection of QTL

The 1 Mb windows that contributed the greatest proportion of genetic variance for each genomic method are shown in Table 1. Using all data from the first 7 generations detected a very large QTL on chromosome 4 at 78 Mb (4_78) using either of the mixture models, and at 4_77 using the models that fitted all loci simultaneously. The effect attributed to this QTL has been shown in other data to be associated with differential expression of the cholecystokinin receptor A (CCKAR) gene [14], which is located around 2 Mb away at ~75.6 Mb. That gene appears to increase orexigenic drive by reducing feedback from the gut to the brain, signaled by CCK [14]. The precise location or nature of the causal mutation responsible for these expression differences is not known. Although it is conceivable that SNPs in 4_78 are in high long-range LD with variants in CCKAR, the 4_78 QTL region on chromosome 4 also includes the LCORL and NCAPG genes, which have been observed to be associated with growth or stature in numerous mammalian GWAS studies [15–18]. So, although this effect could involve long-range LD, or a long-range enhancer acting on CCKAR, it could alternatively be the effect of a different gene, conserved across birds and mammals. Regardless of the cause, a positive genetic correlation has been reported between body weight and yolk weight and between body weight and egg weight for which this QTL is a major contributor [8].

**Table 1:** Comparison of four genomic methods (BayesB, BayesC, BayesC0 and GBLUP) for QTL detection using different generations (G1 to G7) of training data

	BayesB			BayesC			BayesC0		GBLUP	
Training Data	chr_Mb^a^	%Var^b^	*p* >0^c^	chr_Mb	%Var	*p* >0	chr_Mb	%Var	chr_Mb	%Var
G1-G7	4_78	23.7	1.00	4_78	14.4	1.00	4_77	0.62	4_77	0.62
G1	4_78	9.4	0.82	4_78	2.5	0.68	3_110	0.36	3_110	0.32
G2	4_78	6.1	0.73	4_78	6.1	0.73	Z_39	0.31	Z_39	0.32
G3	4_78	13.8	0.64	4_78	3.1	0.77	Z_9	0.32	Z_27	0.35
G4	4_78	22.7	0.98	4_78	4.1	0.90	Z_27	0.54	Z_27	0.58
G5	4_78	10.4	0.77	4_78	1.1	0.50	Z_27	0.35	Z_27	0.36
G6	4_78	7.1	0.77	3_42	3.2	0.93	3_110	0.38	3_110	0.31
G7	Z_23	4.7	0.77	Z_23	3.6	0.77	Z_23	0.32	Z_23	0.34

^a^Localization of the 1 Mb window that explained the largest amount of variance (chromosome_1 Mb window on that chromosome)

^b^percentage of variance explained by that window

^c^proportion of models where this window accounted for more than 0 % of genetic variance

The 4_78 window accounted for 23.7 % of the genetic variance for egg weight when using BayesB, and 14.4 % when using BayesC. In small datasets such as used here, BayesC is expected to result in smaller estimates of variance than BayesB, because BayesC shrinks all SNP effects according to the same variance ratio, whereas BayesB shrinks the effect of each SNP according to the ratio of the residual variance to the unique variance estimate for that SNP. The sampling of the SNP effects variances involves a function of the square of the sample of the SNP effect [19] that results in little shrinkage of large effect loci and considerable shrinkage of small effect loci. In contrast, the two methods that simultaneously fitted all 24,383 equal variance loci using 2,114 animals in the first 7 generations detected the largest QTL 1 Mb upstream at 4_77 and attributed less than 1 % genetic variance to that window.

Using Bayes B for training only on data from any one of the first 7 generations involving at most 360 animals, detected the largest QTL at 4_78 in all but generation 7. Using BayesC detected the largest QTL in 4_78 in all but generations 6 or 7. The estimated effects of the 4_78 QTL were always smaller than obtained with training on all 7 generations, and were never larger for BayesC than for BayesB. The effect of the variance ratio in shrinking SNP effects reduces as the size of the dataset increases [12], and is expected to result in consistent window variances for BayesB and BayesC if the dataset is sufficiently large. In contrast, the two methods that fitted all SNPs never detected the largest effect SNP on chromosome 4 when data from only a single generation was used. Thus, the mixture models were clearly better at detecting large effect QTL than models such as GBLUP, especially in small datasets.

### Accuracy of prediction

The accuracies of prediction of the animals in the most recent generation 8 based on training on data from all the 7 previous generations or from any one of the previous 7 generations are in Table 2. For every method, the most accurate predictions were obtained when training on the much larger dataset comprising all 7 generations. The poorest accuracy was for pedigree based BLUP (PBLUP), which had accuracy of 0.27 for predicting phenotype, while the mixture models had accuracy of 0.57 and outperformed the models that fitted all SNPs, which had an average accuracy of 0.50.

**Table 2:** Accuracy of prediction of phenotypes in generation 8 based on training in all seven previous generations (G1-G7) or any one of the previous 7 generations for pedigree BLUP (PBLUP) and four genomic methods (BayesB, BayesC, BayesC0 and GBLUP)

	N	Method of training with validation in generation 8				
Training		PBLUP^a^	BayesB^b^	BayesC^b^	BayesC0	GBLUP^c^
G1-G7	1,814	0.27	0.60	0.57	0.50	0.50
G1	295	0.10	0.35	0.33	0.19	0.20
G2	323	0.00	0.36	0.36	0.24	0.24
G3	294	0.02	0.49	0.45	0.28	0.28
G4	360	−0.07	0.40	0.30	0.12	0.12
G5	290	−0.01	0.47	0.38	0.32	0.32
G6	252	0.19	0.49	0.44	0.33	0.34
G7	295	0.22	0.48	0.45	0.37	0.37
Average^d^		0.06	0.43	0.39	0.26	0.27

^a^Pedigree-based BLUP

^b^Mixture models assumed the fraction of SNPs with 0 effect (π) of 0.99

^c^GBLUP was fitted as BayesC0 with genetic and residual scale factors having 100° of freedom

^d^Average of the 7 individual generation results

Training on only a single generation to predict generation 8 had no predictive power for PBLUP, unless the training generation was only 1 or 2 generations distant from generation 8 (i.e. generation 6 or 7). Averaged across all 7 individual training generations, PBLUP resulted in accuracy of 0.06, which would account for less than 1 % of phenotypic variance. For the genomic methods, accuracy decreased with distance between the training and validation population, confirming published results [20], but all methods had some predictive power in generation 8 based on training in generation 1. On average, training in any one generation had accuracy of 0.43 for BayesB and 0.39 for BayesC, compared to less than 0.27 for the models that fitted all SNPs with an equal variance ratio. These findings confirm previous studies reporting that BayesB outperforms GBLUP for genomic prediction in the presence of large effect QTL using both simulated [21] and real data [22].

### Persistence of the accuracy in genomic predictions

One of the appeals of genomic selection is its application to traits that are difficult or costly to measure. Ideally, one would train in a suitable population of individuals with phenotypic and genomic information, and then apply the resulting prediction equation in successive generations of selection candidates without investing the time and effort for ongoing phenotypic measurement. The accuracy of this scenario for this case with a QTL of large effect is demonstrated in Table 3, where training was undertaken using only 295 animals from generation 1 and accuracies of prediction were obtained for each successive generation. Not surprisingly, the predictive ability of PBLUP was greatest in the immediate next generation after training but had no real predictive power in subsequent generations. The mixture models showed the most promising results, with BayesB and BayesC accounting for an average of 10 and 9 % phenotypic variance across generations 2 to 8. The models that fitted all SNPs accounted for on average 6 % of variance. In the last validation generation, 7 generations distant from training, the mixture models were still accounting for 10 % of phenotypic variation, whereas the models fitting all SNPs accounted for only 4 %.

**Table 3:** Accuracy of predicting phenotype in each successive generation based on training on generation 1 for pedigree BLUP (PBLUP) and four genomic methods (BayesB, BayesC, BayesC0 and GBLUP)

Validation generation	Method of training in generation 1				
	PBLUP^a^	BayesB^b^	BayesC^b^	BayesC0	GBLUP^c^
G2	0.21	0.36	0.34	0.32	0.31
G3	0.02	0.22	0.22	0.17	0.17
G4	0.12	0.32	0.31	0.28	0.28
G5	0.01	0.27	0.25	0.20	0.20
G6	0.12	0.42	0.39	0.35	0.35
G7	−0.05	0.28	0.28	0.21	0.21
G8	0.10	0.35	0.33	0.19	0.20
Average^d^	0.07	0.32	0.30	0.25	0.25

^a^Pedigree-based BLUP

^b^Mixture models assumed the fraction of SNPs with 0 effect (π) of 0.99

^c^GBLUP was fitted as BayesC0 with genetic and residual scale factors having 100° of freedom

^d^Average of the 7 individual generation results

### Contribution of the largest QTL to the accuracy of genomic prediction

Genes with large effects can obviously contribute a greater than average contribution to predictive ability. Genomic predictions based on training in generation 1 using all SNPs but computing GEBV only using estimates of effects for SNPs in the window that contributed the most variance, as identified in the second row of Table 1, were used to compute the persistence of accuracy over generations, similar to what was done for whole genome GEBV in Table 3. The results are in Table 4 and show that 7 % of phenotypic variance can be predicted by the largest QTL region when using BayesB or BayesC and training on the 295 animals from generation 1, whereas the models that fitted all SNPs had no predictive ability. The latter is not surprising, as Table 1 had demonstrated that these methods resulted in inconsistent locations and small estimates of the effect of the largest QTL. The predictions using BayesB and BayesC indicate a slight trend of increasing accuracy, reflecting the fact that the frequency of the chromosome 4 QTL appears to be increasing slightly over these successive generations [8].

**Table 4:** Accuracy of predicting phenotype in each successive generation when using only the marker effect estimates from the top genomic 1 Mb window based on training in generation 1 for four genomic methods (BayesB, BayesC, BayesC0 and GBLUP)

Validation generation	Method of training in generation 1			
	BayesB^a^	BayesC^a^	BayesC0	GBLUP^b^
G2	0.25	0.23	−0.06	−0.03
G3	0.23	0.19	0.08	0.08
G4	0.27	0.26	−0.03	−0.10
G5	0.29	0.28	−0.13	−0.15
G6	0.31	0.30	0.11	0.13
G7	0.30	0.29	−0.03	−0.02
G8	0.34	0.34	0.00	0.00
Average	0.28	0.27	−0.01	−0.01

^a^ Mixture models assumed the fraction of SNP with 0 effect (π) of 0.99

^b^ GBLUP was fitted as BayesC0 with genetic and residual scale factors having 100 df

### Bias in SNP effects estimation

The within generation estimates of SNP effects from different training data sets using BayesB were compared to fixed effect estimates from a single marker model, fitting an animal model using full pedigree to correct for family relationships in ASReml. For every data set, Table 5 demonstrates that the single marker model estimated larger SNP effects than BayesB. However, as more data were used (all 7 generations) the shrinkage of the BayesB estimate was reduced. These results are consistent with the downward bias (regression coefficient larger than 1) when predicting generation 8 phenotypes using predicted merit based upon SNP estimates from the 1 Mb window with the largest effects using either the single SNP animal model or BayesB. We therefore conclude that in this data there is no evidence for overestimation of the largest effect using the single SNP animal model and therefore, in contrast to the claims of [4], no evidence of overestimation using BayesB.

**Table 5:** Estimates of substitution effects and regression coefficients for predicting generation 8 phenotypes from training in ancestral generations (G1 to G7) for SNP rs14491030 using BayesB or a single SNP model in ASReml

Training generation	BayesB^a^		Single SNP animal model^b^	
	Effect	Regression^c^	Effect	Regression^c^
G1-G7	2.55	1.55	3.05	1.53
G1	0.51	2.51	2.62	1.78
G2	1.13	4.05	2.64	1.77
G3	1.54	2.98	2.83	1.65
G4	2.64	1.77	2.96	1.58
G5	1.69	2.72	3.29	1.42
G6	1.51	2.33	4.05	1.15
G7	0.46	1.74	3.81	1.23
Average^d^	1.50	2.46	3.16	1.51

^a^The effect of the most significant marker in the 1 Mb window with the largest variance

^b^Most significant marker fitted as a fixed effect in an animal model using ASReml

^c^Regression of hatch-adjusted phenotype on predicted merit using the estimate of the SNP effect

^d^Average of the 7 individual generation results

## Conclusions

The mixture models known as BayesB or BayesC more accurately identified the QTL region than the two models that fitted all markers, BayesC0 or the SNP equivalent of GBLUP. Thus, mixture models should be preferred for traits with large effect QTL over models such as GBLUP, which fit all SNP effects assuming equal SNP variance for both genome wide association studies and for genomic prediction. We anticipate that the same finding will be true for models that include information on non-genotyped individuals. That is, single-step mixture models such as proposed in Fernando et al. [23] are expected to outperform single-step GBLUP [24] in the presence of large-effect QTL, until such a time that SNPs comprising randomly-chosen genome-wide markers can be replaced with SNPs in high or perfect linkage disequilibrium with causal mutations that are fitted as fixed effects or weighted according to their contribution to genetic variance.
